# Liposomes can both enhance or reduce drugs penetration through the skin

**DOI:** 10.1038/s41598-018-31693-y

**Published:** 2018-09-05

**Authors:** Ma. F. Peralta, Ma. L. Guzmán, A. P. Pérez, G. A. Apezteguia, Ma. L. Fórmica, E. L. Romero, Ma. E. Olivera, D. C. Carrer

**Affiliations:** 1 Instituto de Investigación Médica M y M Ferreyra - CONICET- Universidad Nacional de Córdoba, Córdoba, Argentina; 2 UNITEFA - CONICET, Pharmaceutical Sciences Department, School of Chemistry, National University of Córdoba, Córdoba, Argentina; 3 Centro de Investigación y Desarrollo en Nanomedicinas (CIDeN)- Universidad Nacional de Quilmes, Bernal, Argentina

## Abstract

The adequate formulation of topical vehicles to treat skin diseases is particularly complex. A desirable formulation should enhance the accumulation of the active drugs in the target tissue (the skin), while avoiding the penetration enhancement to be so large that the drugs reach the systemic circulation in toxic amounts. We have evaluated the transcutaneous penetration of three drugs chosen for their widely variable physicochemical properties: Amphotericin B, Imiquimod and Indole. We incorporated the drugs in fluid or ultra-flexible liposomes. Ultra-flexible liposomes produced enhancement of drug penetration into/through human skin in all cases in comparison with fluid liposomes without detergent, regardless of drug molecular weight. At the same time, our results indicate that liposomes can impede the transcutaneous penetration of molecules, in particular small ones.

## Introduction

Liposomes contain three distinct environments for drugs to dissolve in: the water-lipid interface, the hydrophobic core, and the aqueous interior. As such, they are useful to dissolve/carry hydrophobic, hydrophilic and amphiphilic drugs and antioxidants. This is important since many drugs of therapeutical interest have poor water solubilities and/or form unstable solutions.

In the field of topical vehicles for dermal/transdermal delivery, ultra-flexible liposomes are often considered the vehicle of choice, mainly because of their high performance as transdermal penetration enhancers and their good stability in suspension^1–6^. Ultra-flexible liposomes are composed by a mixture of lipids with low phase transition temperatures and an appropriate amount of a detergent. The detergent acts as a membrane destabilizer, producing an increase in membrane deformability. Ultra-flexible liposomes high deformability has been proposed to be the cause of their ability to penetrate the skin and even allow for proteins to reach systemic circulation^7–13^. However, in the case of diseases where the target tissue is the skin and the active drugs are very toxic to the host other organs, it is of high interest to know if ultra-flexible liposomes would be the best choice for treatment. In the case of Cutaneous Leishmaniasis (CL) for example, the goal of a topical formulation would be to reach the dermis, where the parasites accumulate, but avoid an extensive distribution into the systemic circulation.

The effect of the drugs on (initially) highly flexible liposomes is rarely measured or taken into account. Also, the penetration into/through the skin of a drug *per se* depends on many factors (size, polarity, charge, concentration in the formulation, temperature, solubility, etc.), and it is almost impossible to predict, based on a molecule’s structure and known physical properties, whether it will penetrate the skin or not, at which rate and to which extent.

We initially hypothesized that a change in membrane fluidity produced by the incorporation of a drug in ultra-flexible liposomes would correlate with a change in penetration. We measured the skin penetration of three drugs that possess very different physicochemical properties. AmphotericinB (AmB) is one of the most effective drugs for systemic treatment of Leishmaniasis, especially in its liposomal formulation, which diminishes its (very serious) side effects. AmB is an amphipathic polyene molecule, with a MW of 924.09 g/mol; it has no formal charge; its water solubility is poor, approx. 750 mg/l at 25 °C; its octanol/water partition coefficient logP is 0.8; it has a high affinity for membranes and it tends to form aggregates in water^14,15^. Imiquimod (Imiq) is a nitric oxide producer and an immune modulator that has been shown to have a good *in vitro* activity against some varieties of *Leishmania*^16^. Compared to AmB, it has an intermediate size and complexity. Its MW is 240.31 g/mol; it has no formal charge; its water solubility is poor, approx. 247 mg/l at 25 °C; its logP is 2.7^17^. Indole (Ind), to the best of our knowledge, has not yet been tried against *Leishmania*. Some of its derivatives show promising results against some forms of the parasite^18^. Ind has been proven to be an important modulator of the immune system^19^, and we have obtained promising results against *L. Amazonensis in vitro* (Peralta *et al*., unpublished results). Ind is an aromatic bicyclic compound; it has a small size and complexity compared to AmB and Imiq. Its MW is 117.15 g/mol; it has no formal charge; its water solubility is good, approx. 3,560 mg/l at 25 °C; its logP is 2.14^20^. In summary, we have studied the penetration of one big, complex, insoluble drug (AmB); one medium-sized, relatively simple, insoluble drug (Imiq) and one small, simple, soluble drug (Ind).

We incorporated the drugs either in SoyPhosphatidylcholine:NaChol (SPC:NaChol) (ultra-flexible) or SPC (fluid but not ultra-flexible) liposomes. We measured the penetration both of lipids and of drugs from these systems into mouse skin *in vivo* and human skin *in vitro* respectively; evaluated the effect of the drugs on the liposomes deformability; and measured drug penetration from ultra-flexible or fluid liposomes.

## Results

### Liposomes show size stability

**Figure 1:**
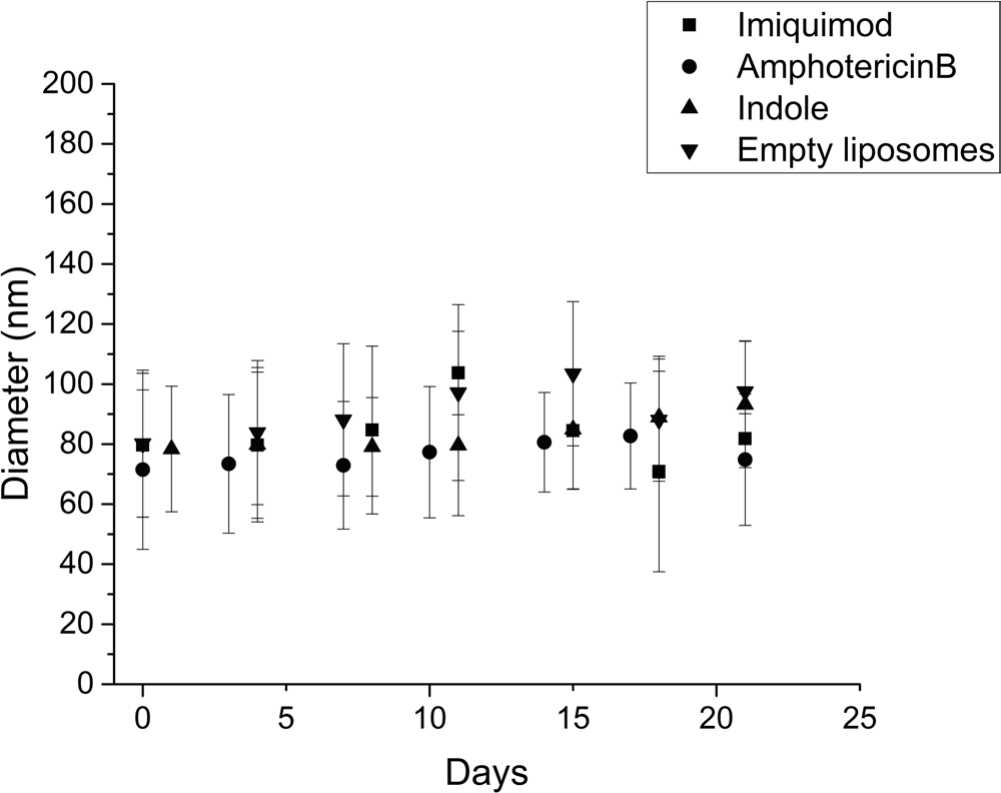
Diameter of liposomes containing different drugs as a function of time. Samples were prepared on day 0, kept at 4 °C and measured every two or three days by Dynamic Light Scattering. The results are the mean of measurements from three independent samples.

After extrusion, empty liposomes showed an average diameter of 100 nm. The addition of Imiq, AmB and Ind in a mol ratio of 10:1 lipid:drug did not significantly change the diameter of liposomes immediately after extrusion, nor a second population with a different size appeared in the samples (Fig. 1). All liposomes maintained their diameter over the assayed period of 21 days.

### Indole increases liposomes deformability

**Figure 2:**
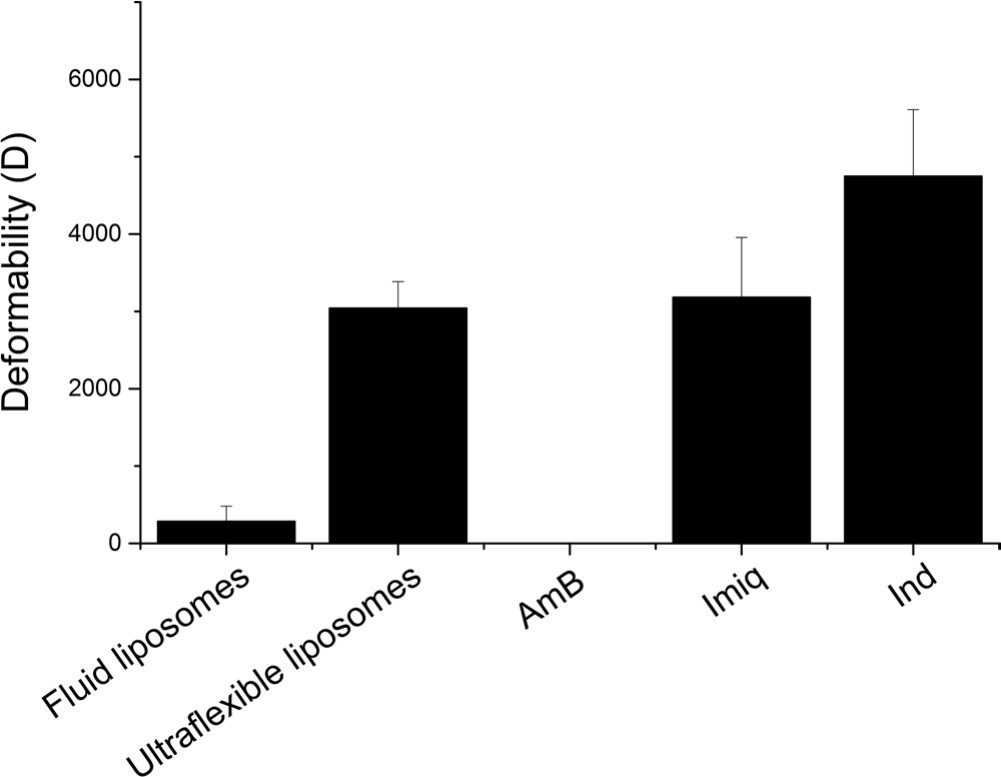
Liposomes deformability measured by extrusion. Deformability was calculated according to D = J × (rv/rp)^2^, were J is the phospholipids flux, rv is the vesicle size after the assay and rp is the pore membrane size. Results are shown for empty fluid and ultra-flexible liposomes, and for ultra-flexible liposomes containing the different drugs.

Deformability of the liposomes (empty fluid and ultra-flexible, ultra-flexible with drugs at 10:1 mol:mol lipid drug relation) is shown in Fig. 2. NaChol produces an important increase on the deformability of the membrane^21^. Ind increases the deformability of the ultra-flexible liposome membrane, while the incorporation of Imiq does not produce a significant difference (p ≤ 0.05). This parameter could not be measured on ultra-flexible liposomes containing AmB since the dispersion did not pass through the membrane at the pressure used (1 MPa).

### *In vitro* human skin penetration/retention varies greatly

The methods used for extraction and quantification of drugs in human skin were assessed for linearity, precision and accuracy (Supplementary Table S2). The methods were accurate, precise and reproducible. Variation coefficients were less than 15% in all cases. Recovery percentages are also shown in Supplementary Table S2.

All the assayed drugs, from liposomal suspensions as well as from aqueous solutions/suspensions, reached the dermis. However, Kp (permeability coefficient) can only be determined when the assayed drug reaches the receptor compartment of the Franz cell in measurable amounts. This condition was only met in the samples incubated with Ind.

**Figure 3:**
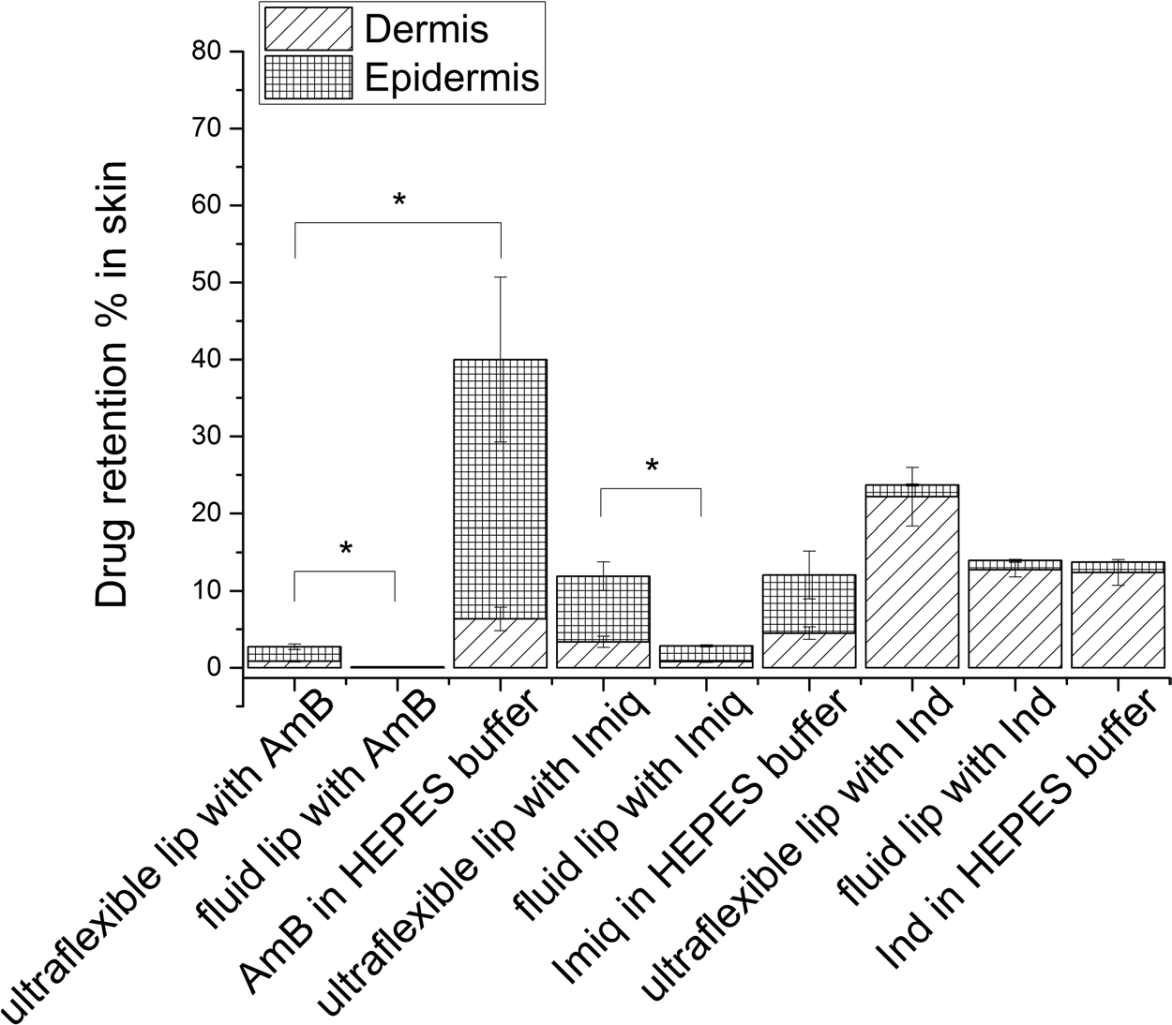
Drugs retention in human epidermis and dermis *in vitro*. Drug retention is quantified as percentage of donor formulation (initial amount). Statistical analysis was performed with ANOVA, P ≤ 0.05.

Figure 3 shows the amount of drug recovered in the different skin layers after 24 hs incubation. As can be observed, when Ind penetration was assessed from ultra-flexible liposomes suspensions, its skin concentration was higher in comparison with its reference solution, with most of the drug being found in the dermis (1.5% of the total amount applied in epidermis (E) and 22.2% in dermis (D)). Ind from fluid liposomes suspensions penetrated in a lower proportion in comparison with Ind in ultra-flexible liposomes suspensions. The permeability coefficient for Ind from ultra-flexible liposomes suspensions was 55.4 ± 0.4 E^−05^ cm^−1^, while Kp for Ind in HEPES was 63.4 ± 0.3 E^−05^ cm^−1^. Ind from fluid liposomes suspensions reached the receptor medium only in the last hour of the assay test, and so it was not possible to calculate Kp in this case. These Kp values indicates that Ind in buffer reaches the receiving compartment in larger amounts compared with ultra-flexible and fluid liposomes suspensions.

When incorporated in ultra-flexible liposomes suspensions, Imiq penetrated moderately into the skin, with most of the drug being found in the epidermis (8.5%) and a significant amount found in the dermis (3.4%) (Fig. 3). The skin penetration percentage of Imiq from ultra-flexible liposomes suspensions was not significantly different from its buffer suspension; however both occurred in a higher proportion in comparison with fluid liposomes suspensions. Since Imiq did not reach the receptor compartment, it was not possible to calculate Kp.

The penetration of AmB into the skin occurred in the order aqueous suspension» ultra-flexible liposomes suspensions > fluid liposomes suspensions (Fig. 3). AmB penetrated to a significant amount when applied as a suspension in buffer to the surface of the skin (6.4% found in dermis and 33.6% in epidermis). When incorporated into ultra-flexible liposomes suspensions, AmB was mostly retained in the epidermis, with a very small amount found in the dermis (1.9% and 0.9% respectively). From fluid liposomes suspensions it was retained in a very small amount in the epidermis (0.1%) and did not reach the dermis.

**Figure 4:**
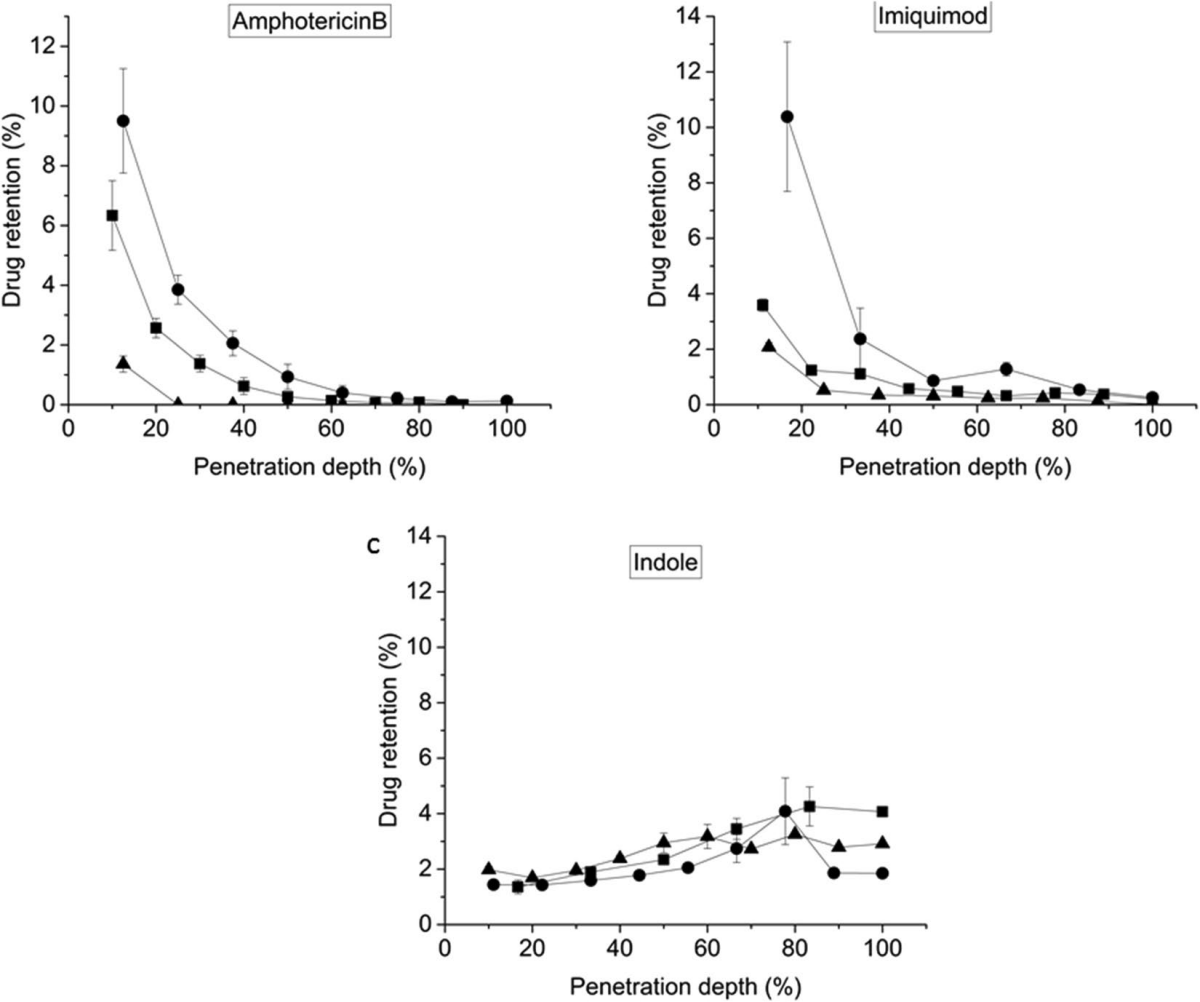
Amount of drug found by HPLC at different layers of human skin *ex-vivo*. Drug amount is shown as percentage amount from donor (formulation put on the skin) versus percentage of skin depth. ●Drug in solution/suspension. ■Drug in ultra-flexible liposomes. ▲Drug in fluid liposomes.

Figure 4 shows the penetration profile of each of the drugs in human skin. Except for the case of Ind, the drugs were found in higher quantities in the first microns of the skin, near the surface. AmB from fluid liposomes suspensions was mostly retained on the surface of the skin (Fig. 4A) and penetrated only to 12.5% of the skin thickness from the surface. However, when incorporated into ultra-flexible liposomes suspensions it was present in measurable amounts up to 80% of the skin thickness, meaning it penetrated the whole epidermis and reached the second third of the dermis. When applied as an aqueous suspension, AmB was found at all depths of the skin and the total amount found in the skin was higher than in the liposomal formulations.

Imiq penetration depth from ultra-flexible liposomes suspensions was highest in the first 46% of the skin thickness. Imiq from aqueous suspension was found mainly in the first 50% of the skin depth. Imiq penetration from fluid liposomes suspensions was the lowest in comparison with both liposomal and aqueous suspension, with most of the drug being found in the first 20% of skin depth (Fig. 4B).

Ind showed a very different profile compared to AmB and Imiq. It was found in higher amounts in the deeper strata of the skin than near the surface. Ultra-flexible liposomes allowed for a higher penetration of Ind in the skin, with most of the drug being found in the deepest parts of the dermis. When incorporated into fluid liposomes suspensions, Ind was retained in similar amounts in all the dermis. Ind in solution shows a peak of retention near the end of the dermis, at 80% of total skin depth, but shows the least amount of drug retained in the skin.

### Liposomes with different drugs penetrate to a similar extent into mouse skin *in vivo*

**Figure 5:**
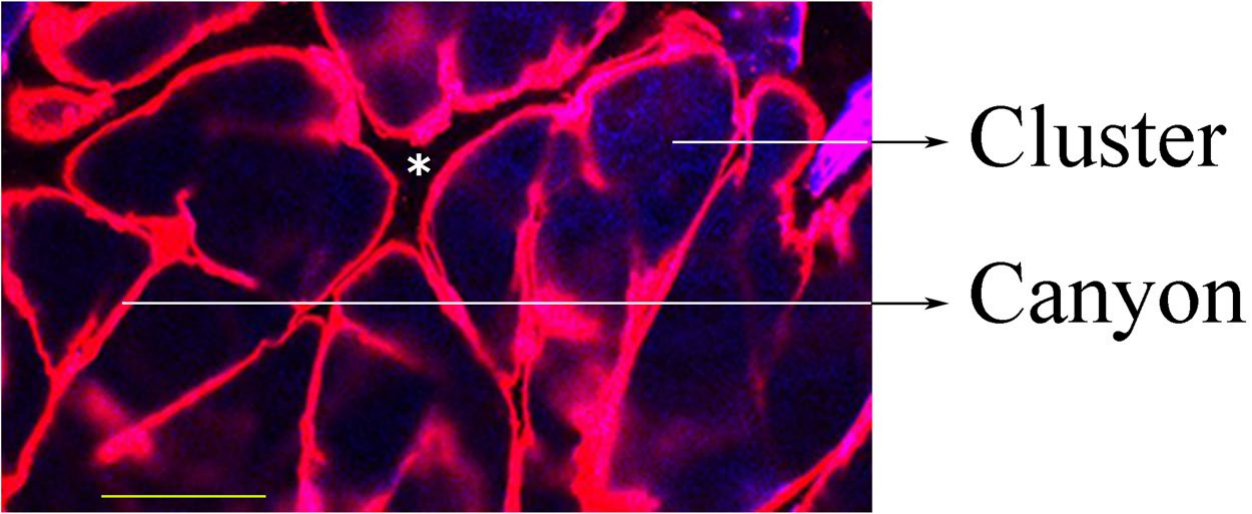
Confocal fluorescence microscopy image of mouse skin. In blue, autofluorescence of the skin (excitation at 405 nm, emission collected at 520–550 nm); in pink, fluorescence of the lipidic dye incorporated in liposomes (excitation at 543 nm, emission collected at 550–630 nm). Two different structures are visible: canyons (invaginations of the stratum corneum) and clusters (groups of corneocytes). The asterisk indicates a wrinkle. The yellow bar indicates 25 μm.

Figure 5 shows a typical fluorescence confocal microscopy image of mouse skin incubated with fluorescent liposomes. The lipidic fluorescent analogue becomes enriched in the furrows (canyons) that separate and envelop the corneocytes clusters.

The epidermis of mice, pigs and apparently also humans is organized in corneocyte columns, with polygonal shapes^9,10,22^. Each column or “cluster” of cells is surrounded and separated from others by a structure called a “canyon”. The canyons seem to be invaginations or extensions of the stratum corneum, which reach the epidermis/dermis interface. They start as a superficial wrinkle and then close, reaching the stratum basal. Canyons are a preferential channel for transepithelial penetration of hydrophobic molecules^10^.

**Figure 6:**
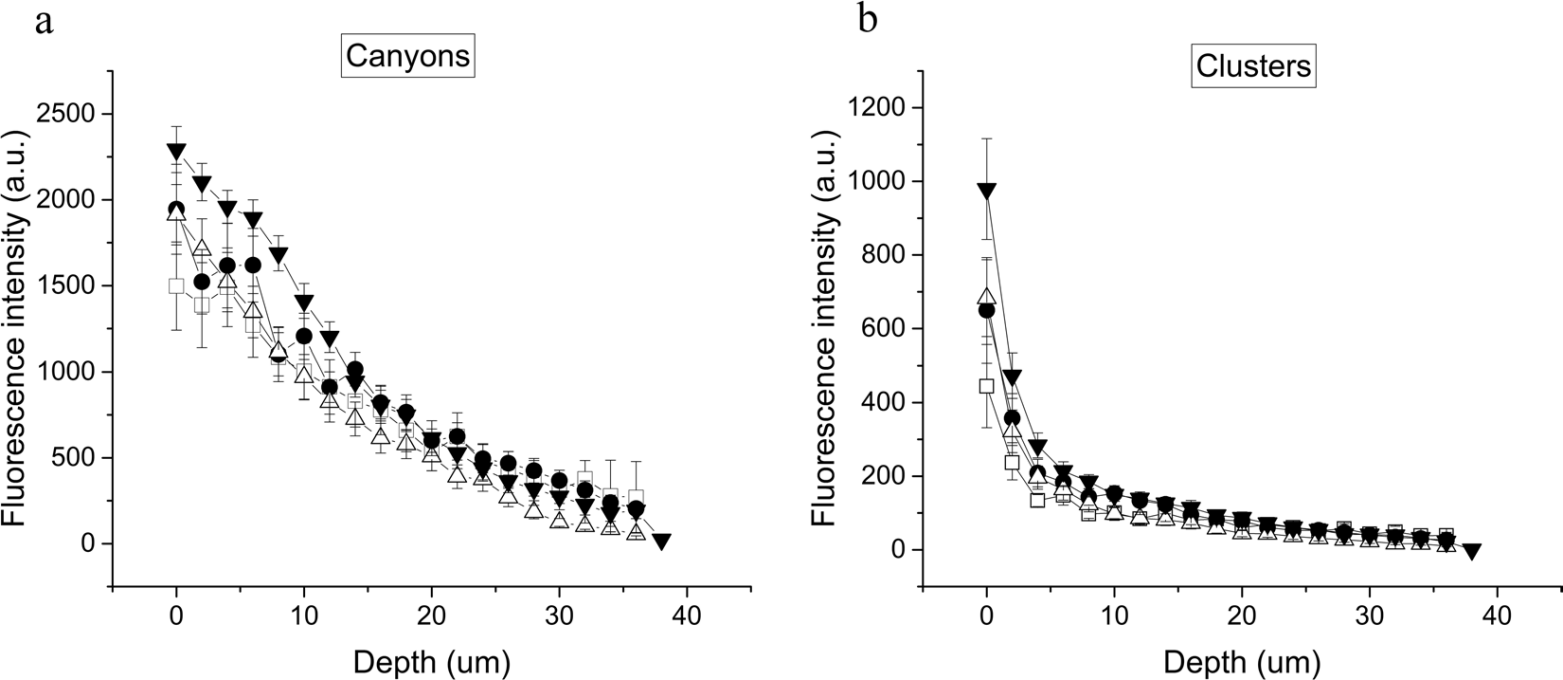
Quantification of ultra-flexible liposomes penetration into mouse skin *in vivo*. Liposomal lipid tracing trough canyons (**a**) and trough clusters (**b**) at different depths in mouse skin. Fluorescence intensity of DiIC18 (fluorescent lipid analogue incorporated into the liposomes) was quantified in micrographs taken by confocal microscopy at planes parallel to the skin surface every two microns. ●Ind-containing ultra-flexible liposomes. Δ Imiq-containing ultra-flexible liposomes. ▲ AmB-containing ultra-flexible liposomes. □ Empty ultra-flexible liposomes.

Figure 6 shows the fluorescence intensity of the lipidic probes incorporated into ultra-flexible liposomes suspensions containing the different drugs at the different depths of the skin, in canyons and clusters respectively. Liposomes penetrate mostly through canyons and in fewer amounts through clusters. Liposomes penetrate until 15 microns through clusters and 30 microns through canyons. Mouse skin has an epidermis of 15 microns thickness, with 6 microns of stratum corneum. Therefore, all dispersions are able to penetrate the epidermis by clusters, and reach the dermis by canyons.

There are no significant differences between formulations containing different drugs, although the liposomal suspension with AmB seems to be more retained in the epidermis than the other formulations.

## Discussion

Pharmaceutical actives frequently have a poor stability, are not well absorbed, are systemically toxic and are hard to transport to the action site. To solve these problems, nanoparticulated carriers like liposomes have won increasing interest^23^. Properly formulated liposomes form suspensions that are stable in size and do not precipitate; liposomes can easily incorporate/aid in the epidermal penetration of drugs of very variable physicochemical properties, they can incorporate lipophilic antioxidants, usually indispensable for formulation stability; lastly, as discussed below, the presence of liposomes allows for a modulation of the drugs penetration/retention into the skin.

The case of skin diseases is particularly complex. A desirable formulation should be able to enhance the accumulation of the active drugs in the target tissue, while avoiding the skin penetration enhancement to be so large that the drugs reach the systemic circulation in amounts that may be toxic. In the particular case of Cutaneous Leishmaniasis, the target tissue is the dermis, and the drugs that show activity against *Leishmania* are extremely toxic to liver, kidneys and bone marrow. Topical formulations containing these drugs are thus of high interest from a clinical perspective.

Drugs penetration through the skin depends on a great variety of factors. One of them is the pathway that drugs take to penetrate into the dermis. The biggest barrier to penetration of the skin is the stratum corneum (SC). This skin structure has a “brick and mortar” organization of corneocytes (the “bricks”, mainly composed of hydrated keratin) and multilamellar layers of ceramides, fatty acids, cholesterol and cholesterol esters^24^ (the “mortar”). These bilayers contain domains in gel and liquid crystal phases and are very hydrophobic, with very long chains, low hydration and high melting temperatures^25,26^. We and others have shown that the epidermis as a whole is organized into clusters or columns of cells, separated from each other by furrows (“canyons”) filled with lipids that traverse the entire epidermis, reaching the basal layer near the dermis. The lipids in these structures have physical properties very similar to those of the stratum corneum, and so we have hypothesized that the canyons may be invaginations of the SC^10,27^. As we show in this manuscript, the lipids from the liposomes penetrate deeper through the canyons than through the cell clusters. It may be possible that some drugs, especially the most hydrophobic drugs, would follow them since the lipidic environment would facilitate lipid-lipid/drug-lipid interactions. Further, the viable layers of the skin (in the clusters, below the SC) can metabolize the drugs, impeding their penetration.

Other important factors that affect drug penetration are: the molecular weight of the molecule, which should be below 600 Da for a good penetration; the solubility of the molecule in oil vs. water (the penetration has been proposed to depend parabolically on logP, with a maximum at a logP of 3^28^). Also the concentration of drug in the donor solution (a larger concentration gradient will produce a larger flux); the charge of the molecule, where charged molecules have a much larger thermodynamic barrier for penetration. An excess of drug in a solution will produce its precipitation in the form of crystals, with a maximum thermodynamic activity at the surface of the skin that will increase the flux but can be irritant and is highly inconvenient for the stability and homogeneity of the pharmaceutical formulation.

The incorporation of liposomes avoids the crystallization of insoluble drugs by dissolving them in a lipid matrix. The physicochemical properties of the vehicle lipids are also important: liquid state vesicles penetrate deeper than gel state liposomes^29^, and highly elastic vesicles facilitate transport across the skin better than rigid or fluid liposomes^13^.

Liposomes of SPC and NaChol are ultra-flexible and its lipidic components are capable of penetrating to the epidermis/dermis interface^10^. Their penetration enhancement capability seems to depend strongly on the deformability of the liposomal membrane^30^.

Ind increases liposome deformability, while Imiq does not have a measurable effect and AmB could not be measured at the concentration used. Other authors, using a much lower concentration of AmB, have measured the deformability of similar liposomes^31^, obtaining D values between 500 and 1600, i.e. a diminished deformability in the presence of the drug. The drug penetration and retention in the skin when incorporated in ultra-flexible liposomes suspensions follow the order Ind > Imiq > AmB. This penetration capability correlates with the deformability results obtained or shown in the literature. However, we found that focusing only in the flexibility of the liposomes was not enough to explain all the results obtained.

In our experiments we did not remove the free drug outside the liposomes. The mechanism of action of liposomes applied to the skin remains a matter of debate. Some authors claim that the liposomes can act as carriers, penetrating intact through the skin, while others propose that liposomes act as penetration enhancers, changing the skin physical properties in a way that facilitates its penetration by the drugs. Honeywell-Nguyen and Bouwstra propose that elastic vesicles carry vesicle-bound drugs into the SC by a fast partitioning, and then the vesicles remain in the SC while the drug continues to the deeper skin layers^32^. Others found that liposomes modulate the penetration not only of the entrapped drugs but also of non-entrapped drug^33,34^. Subongkot *et al*. propose that vesicles could traverse the skin intact through follicular channels^35^. Our experience leads us to support a version of the proposal by Honeywell-Nguyen and Bowstra, namely that liposomes, once applied on the skin, rupture and diffuse as a lipidic mixture that acts as a penetration enhancer. Follicular channels may be a way of penetration for intact liposomes, but at least in the case of humans it would not be a major way of penetration. The liposomes are able to modulate the penetration of the drugs, and their physical characteristics and composition are very important, but they do not penetrate the skin as the original vesicles that we prepare. In this scenario, it is not necessary to remove the free drug, since once the formulation interacts with the skin, all molecules present mix with those of the SC, and the fluidization of the skin lipid membranes produced by the components of the liposomes enhances the penetration of all drug molecules present, regardless of their being initially in the liposomal membrane, the liposomal aqueous interior or the aqueous buffer outside the liposomes.

The *in vivo* experiments in mice show that liposomal lipids penetrate until 20 µm, reaching the dermis, independently of the drug present. So apparently the lipids can influence the drugs penetration but the opposite is not true, at least in this case. In terms of interaction with the skin structure, lipids penetrate more and deeper through canyons than through clusters. This observation coincides with previous reports^10,30^. The added drugs did not change this preference.

In the *in vitro* experiments, we used buffer solutions of the drugs with the intention of having controls for the effect of liposomes on penetration. In the case of Ind, the drug dissolves completely and we get a solution; however, AmB and Imiq have very poor solubilities in water and, at the concentrations used, form suspensions in which the solid particles are surrounded by a layer of saturated solution. The activity of the drugs in the solid particles and in this solution layer is the highest possible^24^, and this would explain why AmB and Imiq in suspension penetrate more than in liposomal formulations, where the drugs are well dissolved and homogeneously dispersed but mixed with the liposomes components, therefore having the same overall concentration but a lower activity. For this reason, it is not possible to directly compare the penetration of the drugs suspensions in buffer with the liposomal samples. The aqueous suspensions are rather extra samples to analyse. Thus we do not know precisely if the liposomes enhance the penetration of AmB and Imiq or not, but they undoubtedly allow for the formulation of stable suspensions containing high concentrations of hydrophobic drugs without the need of using organic solvents. The two drugs in these stable liposomal formulations are able to penetrate to the dermis.

AmB, the largest of the molecules studied, penetrated into the skin best when applied as an aqueous suspension and penetrated very little when applied in fluid liposomes. The amount found in the skin follows the order aqueous suspension ≫ ultra-flexible liposomes suspension > fluid liposomes suspension.

**Figure 7:**
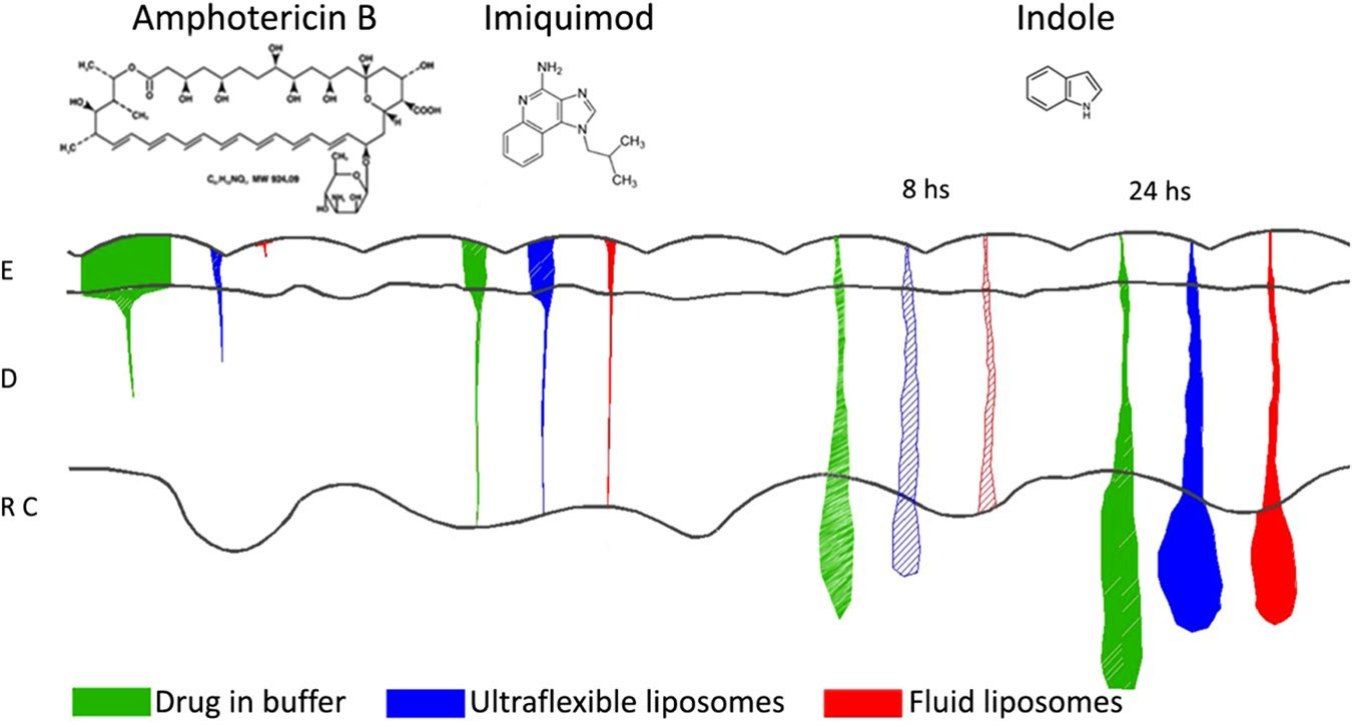
Drug delivery from different vehicles in human skin. ‘Drug in buffer’ stands for a suspension (for AmB and Imiq) and for a solution in HEPES buffer in the case of Indole. 8 and 24 hs indicate incubation times. E: epidermis; D: dermis; R C: receiving compartment. The coloured areas are proportional to the amount of drug found in each stratum of the skin or receiving compartment.

The penetration of Imiq, of intermediate size, showed intermediate penetration results (see Fig. 7). The amount of drug that penetrated into the skin followed the order aqueous suspension ≈ ultra-flexible liposomes suspension > fluid liposomes suspension. In this case we expected to find again that the aqueous suspension penetrated more than the drug in liposomes. However this was not the case. The high flexibility of the liposomes containing NaChol may have balanced the effect of the lowering of the activity of the drug produced by its dilution with lipids and detergent. The fluid liposomes however produced a lowered penetration. Other authors have shown that Imiq penetration is very low, and it depends specifically on the vehicle. Saturated solutions of Imiq in different solvents (different final concentrations) show similar penetration rates^36^.

The penetration of Ind, the smallest drug tested, is strongly determined by its molecular weight. It is well known in the literature that small molecules can penetrate the skin more easily, with a cut-off of approximately 600 g/mole^37,38^. Indeed, Ind was the only molecule that reached the receiving compartment in measurable amounts. It was surprising to find that the presence of liposomes hindered the penetration of Ind as compared to the solution in buffer, even in the case of ultraflexible liposomes. Ultra-flexible liposomes allowed for a larger amount of drug to be retained in the skin compared to the aqueous solution (26% of Ind from liposomal suspension against 13.4% of Ind in buffer). 57% of the applied drug in buffer was found in the receiving compartment of Franz cells after 24 hs, while from ultra-flexible and fluid liposomes suspensions 50% and 32% were found in this compartment respectively. Also the kinetics for drug penetration was different for each formulation. For example, after 8 hs incubation, the amount of Ind that reached the receiving compartment was 27%, 14% or 0% with buffer solution, ultra-flexible liposomes and fluid vesicles respectively (Fig. 7). Thus, liposomes, including ultraflexible liposomes, slowed down Ind penetration.

This retention of Ind in the skin may be produced by molecular interactions (van der Waals, cation – π interactions and hydrogen bonding^39^) occurring between the lipids from the liposomes, the skin lipids and the drug. If the affinity of the drug to the vesicle lipids is high and the solubility in the SC lipids is low, then the liposomes could slow down the penetration of the drug^32^.

In summary:

- Drugs from ultra-flexible liposomes suspensions penetrate deeper and in larger quantities than from fluid liposomes irrespective of the drug’s molecular weight.
- The penetration of the drugs from ultraflexible liposomes correlates well with the changes in flexibility observed, produced by the inclusion of the drugs.

- Aqueous suspensions of insoluble molecules penetrate better than ultra-flexible liposomes suspensions when the drug is very large (AmB), but similarly to ultra-flexible liposomes suspensions when the drug is of intermediate size (Imiq).
- In the case of a soluble drug with a MW < 600, the drug penetrates more when it is in solution, being able to reach the receiving compartment. Its incorporation into liposomal suspensions hinders and slows down its penetration, in particular, ultra-flexible liposomes allow the drug to become more enriched in the skin.

## Conclusions

Ultra-flexible liposomes enhance the penetration of the three drugs, irrespective of the drug’s size, compared to fluid liposomes (see scheme in Fig. 7). On the other hand, when comparing a small soluble drug in solution (Ind) with the same drug in the presence of liposomes, the effect of liposomes is not to enhance penetration but rather to retain the drug in the skin. We do not know which mechanisms are at play, but ultra-flexible liposomes allow a higher amount of drug to be retained in the skin while fluid liposomes slow down Ind diffusion into the skin. This slowing down of diffusion is also seen on the results from Imiq, where the drug from fluid liposomes penetrates the least into the skin. This information suggests that liposomes can either enhance or reduce skin penetration, depending on the molecular weight of the drug and the liposome composition. This may prove of importance when the intention of the formulation is for the drug to stay in the skin rather than reach the blood stream, which is precisely the case when developing a formulation for topical treatment of a skin disease.

## Materials and Methods

L-α-Phosphatidylcholine of Soy (purity >99%) was purchased from Avanti Polar Lipids, Alabama, USA. Sodium Cholate, Indole, Imiquimod and AmphotericinB were from Sigma Aldrich, St. Louis, USA. SnakeSkin® Dialysis Tubing, 3.5 K MWCO was purchased from Thermo Scientific, USA. Methanol and chloroform were from Emsure, Germany. HEPES was from AppliChem, Germany. All other reagents were of analytical grade. For HPLC measurements, methanol and acetonitrile (Anhedra®) were HPLC quality. Absolute ethanol (Sintorgan®) and dietilamine (Anedra®) were FVpharmaceutical grade. For mobile phases, monobasic potassium phosphate and sodium acetate (Ciccarelli®) pharmaceutical grade were used. All experiments were done with Milli-Q water.

### Liposome preparation

Three different formulations were prepared with the following drugs: AmphotericinB, Imiquimod and Indole. Liposomes of SPC containing NaChol are called “ultra-flexible” due to their very low resistance to out-of-plane deformability^13,40^. Ultra-flexible liposomes of SPC:NaChol:drug in a molar ratio of 10:3:1 were prepared by thin-film hydration. Appropriate amounts of SPC in chloroform:methanol (2:1 v/v) and NaChol in methanol were mixed in a glass flask. Solutions of Imiq and AmB were added in this step. AmB was dissolved in DMSO:methanol (1:9 v/v); Imiq in acetonitrile:HCl (100:1 v/v). Solvents were evaporated by a nitrogen flux followed by vacuum for at least 4 hours. The resultant thin-lipid film was hydrated with HEPES buffer 30 mM, pH 7.4 up to a final lipid concentration of 50 mg/ml. This liposomal dispersion concentration was selected for all the following experimental sections. Ind solution was added in the hydration step. Liposomal dispersions were extruded 17 times through a 100 nm pore polycarbonate membrane using a 1 ml extruder (Avanti Polar Lipids, Inc., Alabama, USA) at room temperature. Free drug outside the liposomes was not removed. See the Discussion for an explanation of this choice of method.

### Liposome size and physical stability

All liposomal dispersions were stored at 4 °C and the average liposome diameter was measured every two days for 21 days by Dynamic Light Scattering (Submicron Particle Size NICOMP^TM^ 380, California, USA). For these measurements, samples were diluted in HEPES buffer at a final concentration of 1.6 mg/ml of lipid. Experiments were performed three times independently.

### Deformability measurements

Deformability of liposomes was determined according to Van der Bergh *et al*.^41^. This method is based on the fact that membrane liposome deformability is proportional to its penetration capability through a permeability barrier. An aliquot of 2 ml of each of the drug’s liposomal formulations was extruded (Thermobarrel, Northern Lipids, Burnaby, Canada) at room temperature through 50 nm pore size polycarbonate filters, at a constant pressure of 1 MPa applied with external N_2_. The extruded volume was collected every minute for 15 minutes and phospholipids from liposomes were quantified in each fraction by a colorimetric assay^42^.

Deformability was calculated according to eqn (1):

1 $${\rm{D}}={\rm{J}}\,\times {(\mathrm{rv}/\mathrm{rp})}^{{\rm{2}}}$$

where J is the phospholipids flux (area from phospholipid percentage vs. time plot), rv is the vesicle diameter after the assay (measured by Dynamic Light Scattering with a NanoZsizer) and rp is the pore membrane size (50 nm). Ultra-flexible liposomes without the addition of drugs (empty liposomes) and liposomes without detergent (fluid liposomes) were used as references. Experiments were performed three times independently.

### *In vitro* human skin penetration

In order to evaluate the skin penetration of AmB, Imiq and Ind, assays were performed through abdominal human skin portions in diffusive Franz-type cells (N = 5, i.e. skin from 5 different people) at 36 °C. Human skin was obtained by a plastic surgeon during dermolipectomy surgeries at a local hospital. We worked with discard skin material (procedure approved by Hospital Privado Health Ethics Committee HP 4-158). All experiments were performed in accordance with Consejo de Evaluación Ética de la Investigación de la Salud, CoEIS (Health Ministry of the Province of Córdoba) guidelines and regulations and in compliance with Provincial Law 9694. No information concerning patient’s identity was disclosed to us at any stage, either in coded or uncoded form. The Hospital Privado Health Ethics Committee waived the need to obtain informed consent for the use of these samples. All samples were entirely discarded after analysis. This research is therefore not considered as human subject research. For the assays, the skin was separated from the underlying fat tissue with a scalpel. Skin portions were stored at −20 °C no longer than 30 days. Before assaying, skin pieces were thawed at room temperature. A volume of 180 µl of each dispersion was added on the skin in the donor compartment, with an exposed area of 1.3 cm^2^. Drugs were loaded in ultra-flexible liposomes suspensions. Fluid liposomes and HEPES buffer solutions (for Ind) or suspensions (for AmB and Imiq) at equivalent concentrations were used for comparison. In order to reach sink conditions, the receptor compartment (16 mL) was filled with phosphate buffered saline (PBS) pH 7.4 containing sodium 1% lauryl sulphate for experiments with AmB and Ind and 10% ethanol for the experiments with Imiq^43^.

To determine drug permeation rate through the skin, 1 ml samples from the receptor medium were collected after 1, 2, 3, 4, 5, 6, 7, 8 and 24 hours, with fresh medium replacement. Permeability coefficient K_p_ was determined by eqn (2):

2 $${{\rm{K}}}_{{\rm{p}}}{={\rm{J}}}_{{\rm{ss}}}\,{/{\rm{C}}}_{{\rm{0}}}$$

where C_0_ is the drug initial concentration in the donor compartment (liposomal suspensions on the skin). Stationary drug flux (J_ss_, µg/cm^2^/h) was calculated from the relation between the slope of the quantity of permeated drug vs time plot and the area exposed (1.3 cm^2^)^44^.

After 24 hs, the skin was removed and processed to determine: (1) the amount of drugs retained in the epidermis (E) as well as in the dermis (D) (expressed as percentage of donor drug amount, see eqn (3); (2) the penetration depth (plotted as the percentage of the donor compartment drug amount penetrated vs percentages of the total skin thickness).

3 $$ \% \,{\boldsymbol{retained}}=\frac{\mu {\boldsymbol{g}}\,{\boldsymbol{drug}}\,{\boldsymbol{in}}\,{\boldsymbol{D}}\,{\boldsymbol{or}}\,{\boldsymbol{E}}}{\mu {\boldsymbol{g}}\,{\boldsymbol{drug}}\,{\boldsymbol{in}}\,{\boldsymbol{donor}}\,}\times {\bf{100}}\,$$

After incubation in Franz cells, the skin was rinsed with distilled water, dried with paper and divided in two parts of similar size (parts A and B), which were weighed and processed. Part A was used for the retention assay in order to quantify epidermis and dermis drugs content. For this purpose, specific methodologies were validated according to Supplementary Table S1. Epidermis was separated from dermis by heating at 60 °C in a stove for 5 minutes and peeling off with tweezers. Part B of the skin was used for penetration depth assay: skin was cut with a cryostat every 60 microns and pooled in groups of 6 cuts. Drugs content were determined after their extraction according to Supplementary Table S1.

For penetration depth plots, drug amount is shown as percentage from donor (amount of drug incorporated into each formulation applied on the skin) against the percentage of the total thickness of each of the skin samples studied. The total thickness of the skin samples varied from 2,200 to 3,600 microns.

### Drug extraction from skin and validation of quantification method

In order to quantify each of the drugs retained in the dermis and epidermis, the extraction and quantification were validated by HPLC. Chromatography was performed using a Waters® HPLC system equipped with a 1500 HPLC pump, a 717 auto sampler and a Waters 2996 PDA detector. Data acquisition and processing were performed using Empower® system software. The temperature was maintained with a Waters 1500 series column heater at 25 °C or 38 °C depending on the drug.

Extraction and quantification methods used for each drug and skin portion are summarized in Supplementary Table S1. Linearity, specificity, recovery, precision, accuracy and lower limit of quantification were determined for each method. Samples were prepared as described below. Each value is expressed with its relative standard deviation (%RSD)^45^.

A stock solution of each drug was prepared and then diluted with methanol. AmB was dissolved in DMSO (2.5 mg/ml) and diluted to 5, 10, 50, 100 and 500 µg/ml for epidermis experiments; or to 5, 25, 35, 50, 100 and 250 µg/ml for dermis experiments. Ind was dissolved in HEPES buffer pH 7.4 (1.6 mg/ml) and diluted to 30, 50, 70, 140 and 300 µg/ml for epidermis experiments or to 25, 70, 250, 500, 750 and 1500 µg/ml for dermis experiments. Imiq was dissolved in acetonitrile (1.64 mg/ml) and diluted to 10, 20, 50, 100 and 500 ng/ml for epidermis extractions or to 5, 10, 25, 50 and 250 µg/ml for dermis extractions. An aliquot of 20 µl or 10 µl of these solutions were added to dermis or epidermis segments respectively (skin portions of aprox 0.5 cm^2^), in triplicate. After 2 hs at room temperature, extraction methods described in the Supplementary Table S1 were tested.

### *In vivo* skin penetration in mice

*In vivo* skin penetration of the lipidic components from the liposomal dispersions was studied in female BalbC mice (8 weeks old) by confocal fluorescence microscopy. The aim of this experiment was to follow the lipids of the liposomes dispersions through the different structures of the skin (canyons, clusters, dermis), and to see if incorporation of the different drugs had any effect on penetration depth or preferred way of penetration through the skin. The liposomal suspensions were prepared as before, but with the addition of 1 mol % DiIC18 (a fluorescent lipid analogue) in choloroform:methanol (2:1) to the initial mixture of lipids. The animals were anesthetized (ketamine:xylazine:NaCl 0,9% 5:18:77, 200 µl per animal); the hair on their back was carefully cut with a hair cutting machine and an aliquot of 20 µl of each liposomal suspension was added to 1 sq. cm of exposed skin (see Supplementary Fig. S1). Thereafter the animals were maintained in a temperature (20–24 °C) and humidity (40–70%) controlled room with free access to autoclaved diet and water, subjected to 12 h dark/light cycles.

Twenty four hours after administration, animals were sacrificed by anaesthesia + cervical dislocation. The skin was carefully shaved to eliminate the remaining hair, excised and mounted on a glass slide, without further treatment, with the dermal side in contact with a piece of paper wet with buffer to avoid skin drying during imaging. Confocal images 512 × 512 µm size were taken immediately every two microns with a Confocal Olympus Fluoview 1000 Spectral microscope. The objective used was a PLAPON 60X O NA:1.42. The 543 nm line of a HeNe laser was used to excite the lipidic dye. The fluorescence was collected between 550 and 630 nm. On each animal, two different formulations were tested, with a total of 6 sites of application on each mouse (see Supplementary Fig. S1). Seven experiments were performed for each liposomal dispersion (total n = 21). Ten different locations on each skin sample were investigated. On each image, the area comprising the clusters or the canyons was selected by hand. The fluorescence intensity from each area was measured. Image analysis was performed with Fiji ImageJ.

Animal experimental procedures were approved by the Institutional Committee for Care and Use of Experimental Animals regulation (Instituto de Investigación Médica Mercedes y Martín Ferreyra, Res. N° 013/2017A). The procedures comply with the National Institutes of Health guide for the care and use of Laboratory animals (NIH Publications No. 8023, revised 1978).

### Statistical analysis

Statistical analyses were performed with ANOVA one way, p was ≤0.05 in all cases. For this test, normality was checked by Shapiro Wilks method; variance homogeneity was checked by Levenne test and independency is assured since all samples were freshly prepared. The software Infostat was used. Results are informed as mean and standard error.

## Electronic supplementary material

Supplementary Information

## Data Availability

All information necessary for conduction of experiments is included in this manuscript and/or in the supporting information.
