# Supplementary material for: Liposomes can both enhance or reduce drugs penetration through the skin
Source: Sci Rep. 2018 Sep 5;8:13253. doi: 10.1038/s41598-018-31693-y (PMC6125578; doi:10.1038/s41598-018-31693-y)
Supplement: Supplementary file 1 — Supplementary Information [file 41598_2018_31693_MOESM1_ESM.pdf]

SUPPLEMENTARY INFORMATION for

Liposomes can both enhance or reduce drugs penetration through the skin

Ma. F. Peralta[a¥]; Ma. L. Guzmán[b¥]; A. P. Pérez[c]; G. A. Apezteguia[c]; Ma. L. Fórmica[b]; E. L. Romero[c]; Ma. E. Olivera[b]; D. C. Carrer[a]*

[¥]These authors contributed equally to this work.
[a]Instituto de Investigación Médica M y M Ferreyra - CONICET- Universidad Nacional de Córdoba, Córdoba, Argentina.
[b]UNITEFA - CONICET; Pharmaceutical Sciences Department, School of Chemistry, National University of Córdoba, Córdoba, Argentina.
[c]Nanomedicine Research Program, National University of Quilmes, Quilmes, Argentina.

*Corresponding author: Dolores C. Carrer, dolorescarrer@immf.uncor.edu

Supplementary Table 1

Validated methods for extraction and quantification of each drug for *in vitro* experiments performed in section 2.4 and 2.5: *In vitro* human skin penetration and drug extraction from skin and validation of quantification methods.

Table S1 Methods for drug extraction and quantification from epidermis and dermis on human skin.

Drug	Drug extraction from epidermis	Drug extraction from dermis	HPLC quantification
**AmB**	Extractive solution: 1 ml of methanol.<br><br>1. Vortexing for 1 min, 800 rpm<br>2. Sonication for 10 min<br>3. Incubation at room temperature for 24 hs<br>4. Vortexing for 1 min, 800 rpm<br>5. Sonication for 30 min	Extractive solution: 2 ml of methanol.<br><br>1. Vortexing for 1 min, 800 rpm<br>2. Sonication for 30 min<br>3. Incubation at room temperature for 24 hs<br>4. Vortexing for 1 min, 800 rpm<br>5. Sonication for 30 min<br>6. Vortexing for 1 min, 800 rpm	Column: reverse C18, 250x4 mm, 5 µm packing.<br>Mobile phase: acetonitrile/sodium acetate solution 0.05 M (45/55).<br>Flux: 1 ml/min.<br>Injection volume: 20 µl.<br>Temperature: 25 °C.<br>Wavelength: 405 nm.
**Imiq**	Extractive solution: 1 ml of 7:3 (v/v) MetOH: acetate buffer (pH 4.0, 100 mM).<br><br>1. Vortexing for 1 min, 800 rpm<br>2. Sonication for 10 min<br>3. Incubation at room temperature for 24 hs<br>4. Vortexing for 1 min, 800 rpm<br>5. Sonication for 30 min	Extractive solution: 2 ml of 7:3 (v/v) MeOH: acetate buffer (pH 4.0, 100 mM).<br><br>1. Vortexing for 1 min, 800 rpm<br>2. Sonicationfor 30 min<br>3. Incubation at room temperature for 24 hs<br>4. Vortexing for 1 min, 800 rpm<br>5. Sonication for 30 min<br>6. Vortexing for 1 min, 800 rpm	Column: C8, 125x4 mm, 5 µm packing.<br>Mobile phase: acetonitrile/ sodium acetate solution 100 mM pH4/Diethilamine (30/69.85/0.15 v/v).<br>Flux: 1 ml/min.<br>Injection volume: 20 µl.<br>Temperature: 25 °C.<br>Wavelength: 242 nm.

**Ind**	Extractive solution: 1 ml of methanol.<br><br>1. Vortexing for 1 min, 800 rpm<br>2. Sonication for 10 min<br>3. Incubation at room temperature for 24 hs<br>4. Vortexing for 1 min, 800 rpm<br>5. Sonication for 30 min	Extractive solution: 1 ml of methanol.<br><br>1. Vortexing for 1 min, 800 rpm<br>2. Sonication for 10 min<br>3. Incubation at room temperature for 24 hs<br>4. Vortexing for 1 min, 800 rpm<br>5. Sonication for 30 min	Column: C18, 250x4 mm, 5 µm packing.<br>Mobile phase: phosphate buffer 100 mM pH3/acetonitrile 60:40 v/v.<br>Flux: 1 ml/min.<br>Injection volume: 20 µl.<br>Temperature: 38 °C.<br>Wavelength: 271 nm.

AmB HPLC method was taken from Salerno et al., 2013 [1]; Imiq HPLC method from De Paula et al., 2008 [2] and Ind method was developed for this work [3].

Supplementary Figure 1

Topical application of ultraflexible liposomes suspensions including drugs and DiIC18, for the experiments of section 2.7: In vivo skin penetration in mice.

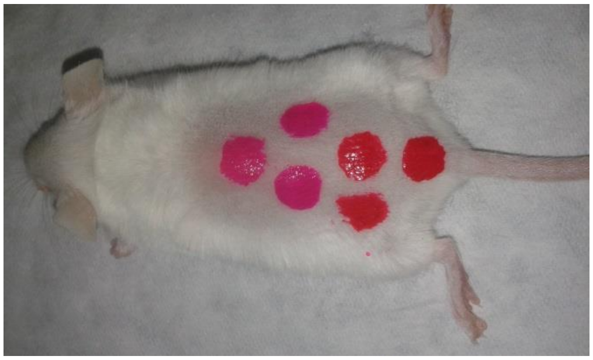

Figure S1 Application of ultraflexible liposomes suspensions including drugs and DiIC18 on the back of anestesized Balb/C mice.

Supplementary Table 2

Results of extraction calibration experiments performed to measure drug retention in human skin.

Table S2 Assessment of linearity, precision and accuracy of the methods used to quantify the amount of each drug in human skin samples. E=epidermis. D=dermis.

	AmB	Ind	Imiq
**Linearity**	0.05 - 5 µg/ml for E<br>0.1 - 5 µg/ml for D	0.3 - 3 µg/ml for E<br>0.5 - 30 µg/ml for D	0.1-5 µg/ml for E and D
**Calibration curve for E**	Y = 93815X-2770<br>R^2 = 0.9993	Y = 58193X-5071.3<br>R^2 = 0.9982	Y = 130480X - 6383.2<br>R^2 = 0.999
**Calibration curve for D**	Y = 88694X-5169.8<br>R^2 = 0.999	Y = 35756X-1271.7<br>R^2 = 0.9964	Y = 116785X-6210.3<br>R^2 = 0.9982
**Recovery percentages**	81.34 ± 2.86 % for E<br>66.9 ± 2.37 % for D	84.21 ± 2.33 % for E<br>55.53 ±2.47 % for D	91.51 ± 1.6 % for E<br>69.17 ± 5.17 % for D
**Lower limit of Quantification**	0.05 µg/ml for E<br>0.1 µg/ml for D	0.3 µg/ml for E<br>0.5 µg/ml for D	0.1 µg/ml for E and D

References

1. Salerno, C. *et al.* Lipid-based microtubes for topical delivery of amphotericin B. *Colloids Surf. B. Biointerfaces* **107,** 160–166 (2013).

2. De Paula, D., Martins, C. A. & Bentley, M. V. L. B. Development and validation of HPLC method for imiquimod determination in skin penetration studies. *Biomed. Chromatogr.* **22,** 1416–1423 (2008).

3. Peralta, M. F.; Formica, M. L.; Palma, S. D.; Carrer, D. C. Development and validation of an HPLC method for quantification of Indole in a liposomal formulation. *Anal. Methods. Submitted*
